# Sun-Spots and Meteorology

**Published:** 1880-06

**Authors:** E. G. Betty


### SUN-SPOTS AND METEOROLOGY.

BY E. G. BETTY, D. D. S.

The following conclusion from a paper contributed to "*Nature*" by H. F. Blanford, seems to add another choice bit of data to that already collected, in the endeavor to definitely determine the Sun's influence upon the Earth's meteorology.

"Between Russia and Western Siberia on the one hand, and the Indo-Malayan region (perhaps including the Chinese region) on the other, there is a reciprocating and cyclical oscillation of atmospheric pressure of such a character that the pressure is at a maximum in Western Siberia and Russia, about the epoch of maximum sun-spots, and in the Indo-Malayan area at that of minimum sun-spots."

The conclusion here apparently leads us to infer that the sun-spots have a direct influence upon the atmospheric pressure in the regions named, and of course the density of the atmosphere will have *its* effect upon the rainfall, and this upon the crops. The failure of the crops in India last year and the partial one in Russia, look very much like a contradiction of the inference.

The study of the Sun's influence upon our Meteorology is certainly one of intense, absorbing interest, and having a very practical bearing upon man's weal and woe.

In general, the temptation to link coincidences together is a seductive one, thus being a fruitful source of error. It should be borne in mind, that at best we know but little, and we should therefore, not be too hasty in forming conclusions.

The Sun is our creator; and it is due to his heat we exist from day to day. The force exerted by the sun is manifested in different forms and ways, so with that in view it is well not to attribute to any particular phenomenon he may display, too powerful an influence upon any one that may happen upon the Earth. They may only be coincident or as in the case of the influence of the sun-spots, the result of some ulterior cause of which we are not cognizant.

A direct effort to connect "disasters in the sun" with mundane events, appeared some years ago in the *Scientific American*. The article was an account of some observations made upon the periodicity of the sun-spots and the periodical changes or disturbances of terrestrial magnetism, as noted by the variation of the needle. So far as is known, about five years and a half are consumed from the epoch of minimum sun-spots to that of maximum, and an equal lapse of time in receding again to the minimum degree. In other words, eleven years are required to complete the cycle, at least so far as our knowledge extends; for we do not know whether the time is constant or whether or no this eleven-year cycle may be but a phase of a cycle so great, that an eternity of time would be required to measure its oscillations.

However, this period corresponds exactly, or nearly so, with the variation of the needle between two points east and west of the geographical north pole.

These two facts, as showing a connection of some kind between solar disturbances and the coincident changes in the Earth's magnitism were so construed by the *Scientific American* writer, as to lead the reader to suppose or rather believe (through an ingenious interpretation of statistics) that the periodical famines of India are due primarily to the regular recurrence of the sun-spots.

Whether this is absolutely true or not, is very difficult to determine, though taken in connection with Mr. Blanford's conclusion, it does seem as though the regular increase and diminution of the sun-spots has somewhat to do with terrestrial meteorology.

'Tis a well established fact that upon every outburst or disturbance in the sun, the needles in every station over the Earth are seen to simultaneously vibrate. This alone is proof enough to demonstrate that the occurence of spots, (which appear to be openings through luminous vapors upon the sun's surface, caused possibly by internal violence) does exert an influence upon terrestrial magnetism. When a large aggregation of spots are observed upon the sun, it has been noticed that his heat has for the time been diminished. It is not impossible but that the changes in temperature together with the friction incident upon the violent movements of matter may cause the evolution of immense quantities of electricity. Under such circumstances, the sun's matter would become magnetised. Disturbances in terrestrial magnetism would necessarily follow. The production of vast quantities of electricity in the sun without magnetization of his matter, would in and of itself disturbe the magnitical condition of the Earth; indeed the late Professor Vaughan in his paper on "The Origin of Worlds" in the "*Popular Science Monthly*" a year ago, introduced Electricity and Magnetism as powerful factors in the formation of cosmical bodies.

Notwithstanding the facts already gleaned, there is yet too little material upon which to base an estimation of the effect sunspots produce upon our meteorology and the growth of crops. The attempt to show an intimate relation between solar affairs and the production of crops upon the Earth and so predict the cost of food, is a problem more seductive and about as easy as the time honored one of perpetual motion.

In the words of Professor Langley: "The attempt has thus far been unsuccessful.

There is hardly any topic on which there is more popular interest, hardly any on which there is more popular error, than this of the supposed influence of the sun on the weather. By means of the study of what Professor Smythe terms the "rainband" in the spectrum, we appear to have lately gained increased facility in predicting local weather changes; but, excepting this comparatively unimportant contribution, studies connected with the sun have as yet done very little for us here, and it seems necessary to say that, as far as prophecy is concerned, none of us as yet are prophets, or more able to tell from our knowledge of the sun what the weather will be next week than what the harvest will be next year."

